# How Visual Perception of the Inside of Things Creates the Impossible Dovetail

**DOI:** 10.1177/2041669520960494

**Published:** 2020-09-30

**Authors:** Vebjørn Ekroll, Rob van Lier

**Affiliations:** Department of Psychosocial Science, University of Bergen, Bergen, Norway; Donders Institute for Brain, Cognition and Behaviour, Radboud University, Nijmegen, The Netherlands

**Keywords:** amodal completion, magic, puzzles, imagery

## Abstract

Here, we consider a well-known wooden puzzle known as the impossible dovetail. We argue that an intriguing form of amodal completion, dealing with spontaneous interpretations of the inside of objects is the key to understanding why people find it difficult to see how the impossible dovetail is indeed possible.

Figure 1 and Movie 1 show a wooden puzzle known as the impossible dovetail (Ringel, 1999; 2012; Wyatt, 1956/2007). Just looking at the puzzle immediately makes one wonder how the upper and lower halves might have been joined and how they may be taken apart. That is, the object triggers reasoning about its causal history (Spröte et al., 2016) as well as the possibilities for actions it affords (Gibson, 1966). But much like a magic trick (Ekroll, 2019; Kuhn, 2019), the assembly and disassembly of the two parts appears impossible.

**Figure 1.**
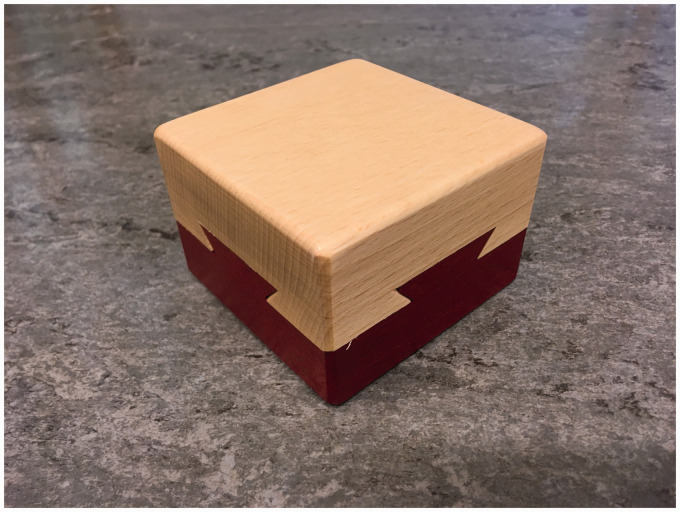
A Wooden Puzzle Known as the Impossible Dovetail. How can the two pieces be taken apart without breaking them? How was it even possible to join them in the first place?

**Movie 1.** Movie illustrating the actual 3D interior shape of the impossible dovetail puzzle.

The work of Gagnier and Shipley (2013, 2016) suggests a simple and intriguing explanation for this. Much like wood has growth rings, many material objects have various forms of internal structures and layers, which are partially visible on the outside and extend into their invisible insides. Investigating people’s intuitions about these invisible insides, Gagnier and Shipley found that they mostly failed to recognize the ambiguity of the visible information at the surface with regard to the invisible internal structure. Furthermore, they found that people have a strong tendency to experience the visible surface structure as extending straight into the object at an angle perpendicular to the surface. Such a tendency or “perceptual heuristic” would neatly explain why people experience it as impossible to join or take apart the two pieces. This is because perpendicular extensions of the visible surface border into the cube (such as the ones shown in Figure 2B) implies the existence of two straight “rails” that are oriented in perpendicular directions relative to each other, so that one rail would prevent free sliding along the other and *vice versa*. Thus, possible layouts of the interior that would actually allow assembly and disassembly of the two parts (such as the one shown in Figure 2D) are excluded from consideration at the very start of the problem-solving process.

**Figure 2.**
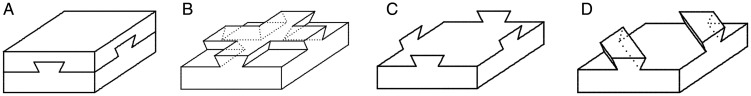
The impossible dovetail puzzle. Presumably, the reason why the dovetail in (A) is experienced as impossible is that people implicitly assume that the interior must be shaped as in panel (B). This implicit assumption may in turn stem from a perceptual heuristic which extrapolates the visible contours at the surface into the object at an angle perpendicular to the surface. The structure visible at the surface (C) is also compatible with the interior shown in (D), which is one of the many possible ways the apparently impossible dovetail may be constructed. Reproduced and adapted from Ringel (1999), with permission.

In an informal experiment exploring the plausibility of this account, we asked 20 participants to draw their immediate impression of the inside of the object shown in Figure 1. They viewed Figure 1 printed on a sheet of paper and were asked to draw what they immediately imagined that the interior would look like if (a) the object was cut in the middle along a horizontal plane, (b) the top part was taken away, and (c) they looked at the bottom part from above. The most frequent response (11 out of 20) was a simple cross, as in Figure 3A. Three additional participants indicated a perpendicular continuation into the object for some distance, but a different (Figure 3B, 2 cases) or absent (Figure 3C, 1 case) further continuation in the central region. Yet another three participants first made drawings indicating that they assumed that the object was an empty shell with a very thin surface. When asked how they would imagine the interior assuming that it was solid rather than hollow, these participants also drew simple crosses, as in Figure 3A. The remaining three participants provided drawings with nonperpendicular elements close to the surface. Thus, to summarize, 70% of the participants demonstrate a preference for perpendicular continuation at least some distance into a solid (nonhollow) object, an additional 15% indicate the same preference after the information was given that the object as solid rather than hollow, and only 15% drew completions involving non-perpendicular completions close to the surface.

**Figure 3.**
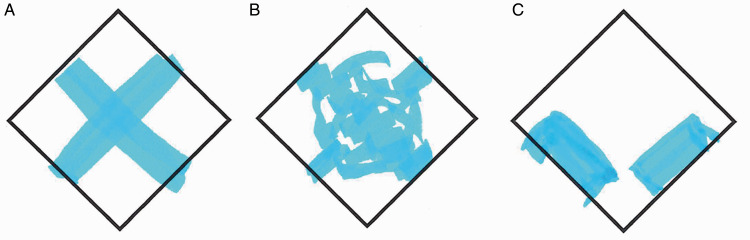
Some Illustrative Examples of the Drawings Made by the Participants in the Experiment. The most frequent type of drawing (11 out of 20) was a simple cross, as in (A). See text for further details.

Previous research on amodal completion strongly suggest that our mental experiences of occluded regions in a visual scene, such as parts of objects occluded behind other objects in the foreground (Kanizsa, 1985; Michotte et al., 1964) or the hidden backsides of things (Ekroll et al., 2018; Michotte & Burke, 1951; Tse, 1999; van Lier, 1999; van Lier & Wagemans, 1999) are shaped by perceptual processes. The idea that perceptual processes and heuristics as sketched above may also determine how we experience the insides of things is therefore not as radical as it may appear at first thought. Indeed, several studies (Gagnier & Shipley, 2013, 2016; Gerbino & Zabai, 2003; Oh, 2020; Vrins et al., 2009) suggest that perceptual heuristics can determine our mental experiences of interior volumes. Gerbino and Zabai (2003) and Vrins et al. (2009), for instance, studied people’s mental experiences of interpenetrating objects similar to the knife-through-arm illusion in Figure 4. In this illusion, which is regularly employed by magicians (Ekroll et al., 2017), people tend to experience the knife as penetrating the arm, rather than the other way around (which is actually the case). Thus, as paradoxical as it may seem, there is good reason to believe that our experiences of the insides of things are at least in part shaped by visual mechanisms. As Koenderink (2015) notes, many objects (e.g., an orange) have skins, which “often hide surprising interiors,” while others (e.g., a wooden sculpture) don’t, as if we can “look right into the interior” (p. 19). The present considerations and observations suggest that the apparent impossibility experienced when viewing the impossible dovetail may be attributed to visual completion mechanisms. The strong preference for experiencing the visible surface structure as extending straight into the object at an angle perpendicular to the surface may reflect a heuristic employed by the visual system that is often a sensible guess, but happens to backfire in the particular case of the impossible dovetail.

**Figure 4.**
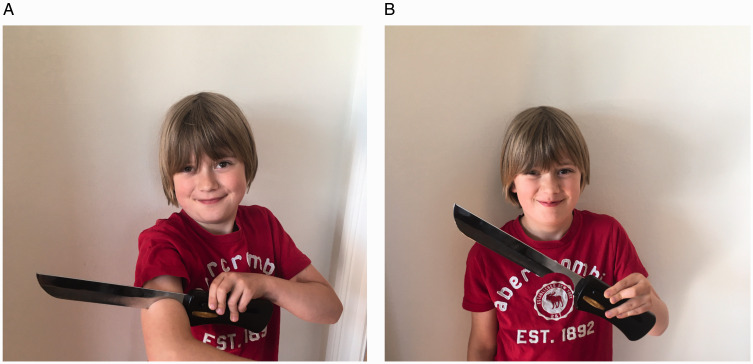
Seeing vs. thinking. In (A) the knife appears to penetrate the arm, but of course, it is the arm that penetrates the blade (B).
